# Prior bariatric surgery and perioperative cardiovascular outcomes following noncardiac surgery in patients with type 2 diabetes mellitus: hint from National Inpatient Sample Database

**DOI:** 10.1186/s12933-020-01084-7

**Published:** 2020-07-06

**Authors:** Jiewen Jin, Zhantao Deng, Lijuan Xu, Hai Li, Pengyuan Zhang, Liehua Liu, Juan Liu, Hedong Han, Zhimin Huang, Xiaopei Cao, Haipeng Xiao, Yanbing Li

**Affiliations:** 1 Department of Endocrinology, The First Affiliated Hospital, Sun Yat-Sen University, No. 58, Zhong Shan Er Lu, Guangzhou, 510080 China; 2 Department of Orthopedics, Guangdong Provincial People’s Hospital, Guangdong Academy of Medical Sciences, School of Medicine, South China University of Technology, Guangzhou, China; 3 Department of Health Statistics, Second Military Medical University, Shanghai, 200433 China

**Keywords:** Bariatric surgery, Noncardiac surgery, Cardiovascular outcomes, Mortality

## Abstract

**Background:**

Both diabetes and obesity are risk factors for perioperative major adverse events. This study aims to evaluate the association between prior bariatric surgery (prior-BS) and perioperative cardiovascular outcomes following noncardiac surgery in patients with type 2 diabetes mellitus (T2DM).

**Methods:**

We used the National Inpatient Sample Database to identify T2DM patients undergoing major noncardiac surgery from 2006 to 2014. The primary outcome was major perioperative adverse cardiovascular and cerebrovascular events (MACCEs), which include death, acute myocardial infarction and acute ischaemic stroke. In-hospital outcomes between patients with prior BS and morbid obesity were compared using unadjusted logistic, multivariable logistic and propensity score matching analyses.

**Results:**

A weighted of 1,526,820 patients diagnosed with T2DM who underwent noncardiac surgery were included. The rates of both prior BS and morbid obesity significantly increased during the study period (P < 0.0001). Patients with prior BS were younger, were more likely to be female, and had lower rates of cardiovascular risk factors but had higher rates of smoking, alcohol abuse, anaemia, prior venous thromboembolism and prior percutaneous coronary intervention. The incidence of MACCEs was 1.01% and 3.25% in patients with prior BS and morbid obesity, respectively. After multivariable adjustment, we found that prior BS was associated with a reduced risk of MACCEs (odds ratio [OR] = 0.71; 95% confidence interval [CI] 0.62–0.81), death (OR = 0.64, 95% CI 0.52–0.78), acute kidney injury (OR = 0.66, 95% CI 0.62–0.70) and acute respiratory failure (OR: 0.46; 95% CI 0.42–0.50).

**Conclusions:**

Prior bariatric surgery in T2DM patients undergoing noncardiac surgery is associated with a lower risk of MACCEs. Prospective studies are needed to verify the benefits of bariatric surgery in patients undergoing noncardiac surgery.

## Background

Worldwide, there are more than 300 million patients who undergo noncardiac surgery each year [1]; major adverse cardiovascular and cerebrovascular events (MACCEs) occur in 1 of every 33 hospitalizations and are a significant source of perioperative morbidity and mortality [2]. Owing to the improvement in surgical and anaesthetic techniques as well as perioperative cardiovascular care [3, 4], the frequency of MACCEs has declined from 3.1 to 2.6% over the last decade [2].

The prevalence of obesity, a well-known risk factor for cardiovascular diseases (CVDs), has been increasing rapidly in the past decade and is expected to reach 18% in men and 21% in women worldwide by 2025; obesity is one of the most important public health conditions worldwide [5]. Obesity greatly increases the difficulty of noncardiac surgery and may attenuate improvements in perioperative outcomes over time [6]. Bariatric surgery is the most effective treatment for morbid obesity and is able to result in long-term weight loss, reduce many obesity-related comorbidities and improve quality of life [7]. A long-term follow-up study revealed a diabetes remission rate of 68% (57% for dyslipidaemia and 61% for hypertension) in obese patients who underwent bariatric surgery [8]. Recent data have shown that compared with conventional medical therapy, the management of patients with type 2 diabetes mellitus (T2DM) and morbid obesity is superior after bariatric surgery [9]. Additionally, a series of randomized trials have shown the superiority of surgery over medical treatment alone not only in achieving improved glycaemic control but also in reducing cardiovascular risk factors [10].

Recently, evidence from the National Inpatient Sample (NIS) of patients aged 45 years who underwent noncardiac surgery showed an unfavourable trend of perioperative MACCEs among subjects with T2DM versus those without T2DM [11]. However, the trend of perioperative MACCEs among patients with T2DM and morbid obesity and the influence of bariatric surgery on MACCEs have not been examined. To address this knowledge gap, we compared the perioperative cardiovascular outcomes after noncardiac surgery among T2DM patients who had undergone bariatric surgery and those with morbid obesity.

## Materials and methods

### Study population

Patients aged ≥ 18 years who were undergoing major noncardiac surgery from 2006 to 2014 were identified from the NIS of the Healthcare Cost and Utilization Project (HCUP). The NIS is an all-payer administrative database that includes data on approximately 8 million hospitalizations per year from a 20% stratified sample of all hospitalizations in the US [12]. Patients were included if they had a principal ICD-9-CM procedure code for a major therapeutic operating room procedure (HCUP Procedure Class 4) during hospitalization. Surgery was categorized through primary Clinical Classifications Software (CCS) procedure codes. Patients who underwent cardiac procedures, cardiac surgery, cardiac transplantation, dental surgery, ophthalmologic surgery, radiation therapy, bone marrow transplantation and non-operating room procedures were excluded. Based on primary CCS procedure codes, major noncardiac surgery was clustered into the following nine subtypes: general, genitourinary, neurosurgery, orthopaedic, otolaryngology, skin and breast, thoracic, vascular surgery and other surgery [13]. The method to identify patients who underwent major noncardiac surgery has been widely used in previous publications. T2DM, prior bariatric surgery (prior BS) and morbid obesity were identified through ICD-9-CM diagnosis codes (Additional file 1: Table S1). We further clarified T2DM as controlled or uncontrolled (Additional file 1: Table S1).

### Patient characteristics

For each hospitalization, baseline characteristics including age, sex, race, comorbidities (including smoking, alcohol abuse, drug abuse, dyslipidaemia, hypertension, coronary artery disease, end stage renal disease, congestive heart failure, chronic lung disease, chronic liver disease, peripheral vascular disorders, malignancy, anaemia, valvular disease, prior venous thromboembolism, prior transient ischaemic attack/stroke, prior myocardial infarction) and prior cardiovascular surgeries (prior percutaneous coronary intervention and prior coronary artery bypass grafting) were identified (Additional file 1: Table S1).

### Outcomes

The primary outcome was the incidence of MACCEs, defined as in-hospital all-cause death, acute myocardial infarction (AMI), or acute ischaemic stroke (AIS). AMI was identified using ICD-9-CM diagnosis codes 410.x1. AIS was identified using ICD-9-CM diagnosis codes 436.x, 437.1, 433.x1, 434.01, 434.11 and 434.91. Other perioperative outcomes, including cardiogenic shock, acute kidney injury and acute respiratory failure, were also identified using corresponding ICD-9-CM diagnosis codes (Additional file 1: Table S1).

### Statistical analysis

According to the HCUP guidance, sampling information was applied to calculate national estimates for all measures [14]. Categorical variables were reported as percentages and compared using Rao–Scott χ^2^ test. Temple trends in rates of prior-BS and morbid obesity were evaluated through Cochran–Armitage trend test. The NIS database was redesigned in 2012 and we updated discharge weights from 2012 to 2014 for accurate estimates of the trend analysis. Temporal changes in BMI categories from 2006 to 2014 were also explored.

Incidence of perioperative cardiovascular outcomes were calculated for patients with prior-BS and morbid obesity. We first performed unadjusted logistic regression analysis to test the differences in rates of perioperative cardiovascular outcomes between the two groups. Furthermore, multivariable logistic regression analysis was conducted to obtain adjusted odds ratios (ORs) for MACCEs and other perioperative cardiovascular outcomes. Covariates in the model included age, sex, race, elective surgery, smoking, alcohol abuse, drug abuse, dyslipidaemia, hypertension, coronary artery disease, end stage renal disease, congestive heart failure, chronic lung disease, chronic liver disease, peripheral vascular disorders, malignancy, anaemia, valvular disease, prior venous thromboembolism, prior transient ischaemic attack/stroke, prior myocardial infarction, prior percutaneous coronary intervention, prior coronary artery bypass grafting, uncontrolled T2DM and surgery type.

In addition, we performed 1:1 greedy propensity score matching analysis without replacement to remove the bias caused by confounding between prior BS and morbid obesity [15]. A logistic regression model incorporating the abovementioned baseline characteristics was used to calculate the propensity scores (probability of prior bariatric surgery). The balance of the baseline covariates before and after propensity score matching were assessed using absolute standardized differences, with a threshold of < 0.1 indicating permissible similarity between the two groups. For the matched sample, conditional logistic regression was used to compare perioperative cardiovascular outcomes. Finally, sensitivity analysis for MACCEs in the propensity score matching analysis was conducted to explore the influence of assumed unmeasured confounders on the robustness of the results [16].

We further performed several sensitivity analyses to test the robustness of results in above analysis. First, diabetes-related complications were adjusted in the multivariable analysis. Second, BMI were adjusted to reduce potential bias caused by BMI inequalities between prior-BS and morbid obesity. Third, we categorized patients in the prior-BS group into two groups (BMI < 35 kg/m^2^ and BMI ≥ 35 kg/m^2^) to explore whether the effects were different in prior-BS patients with and without weight loss. Fourth, patients who underwent BS interventions during the current hospitalization were excluded.

Statistical significance was defined as a P-value < 0.05 on two-tailed testing. Statistical analyses were performed using the SAS software version 9.4 (SAS Institute, Cary, NC).

## Results

### Study population

**Fig. 1:**
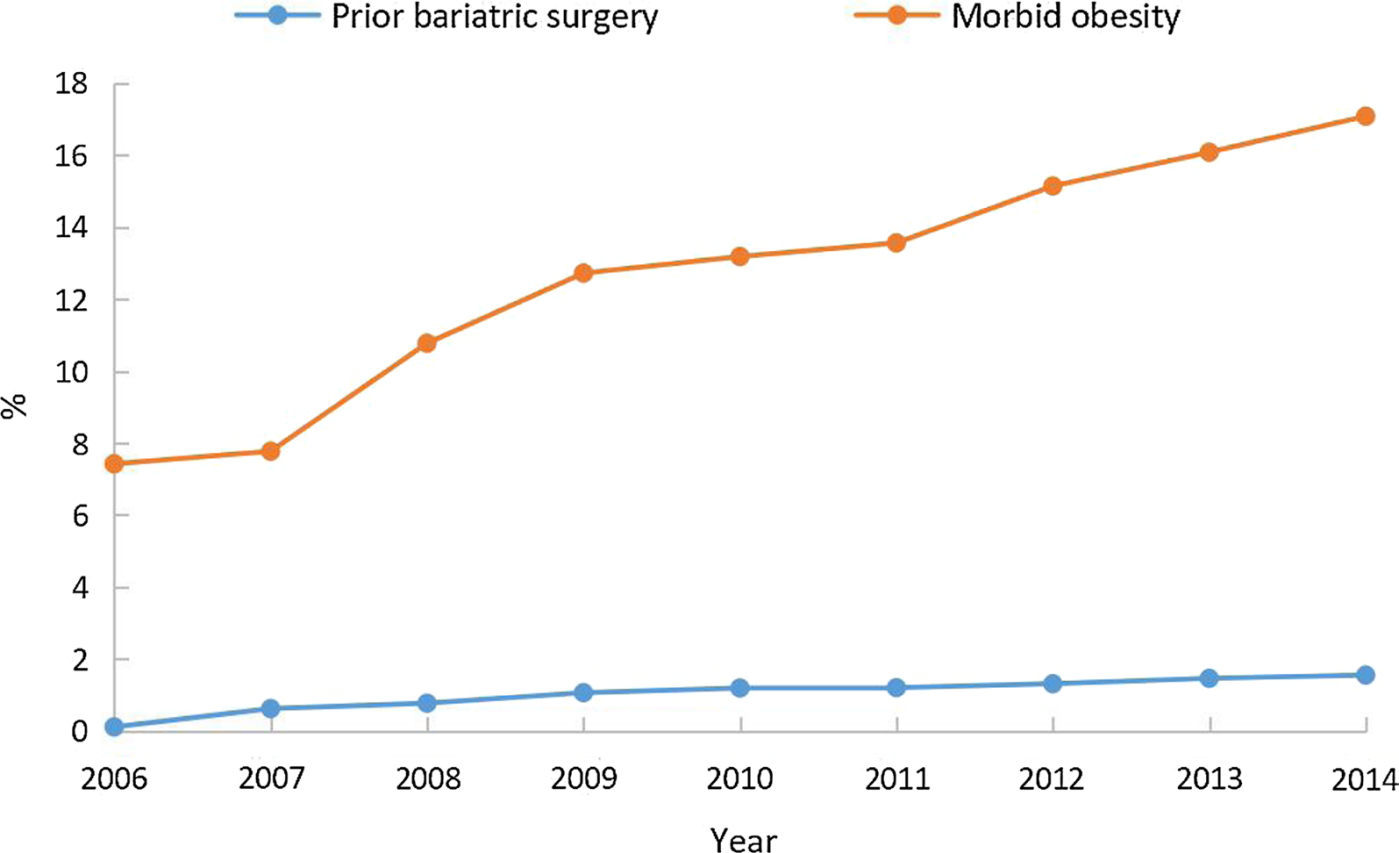
Rate of prior bariatric surgery and morbid obesity in patients with type 2 diabetes mellitus over time

**Fig. 2:**
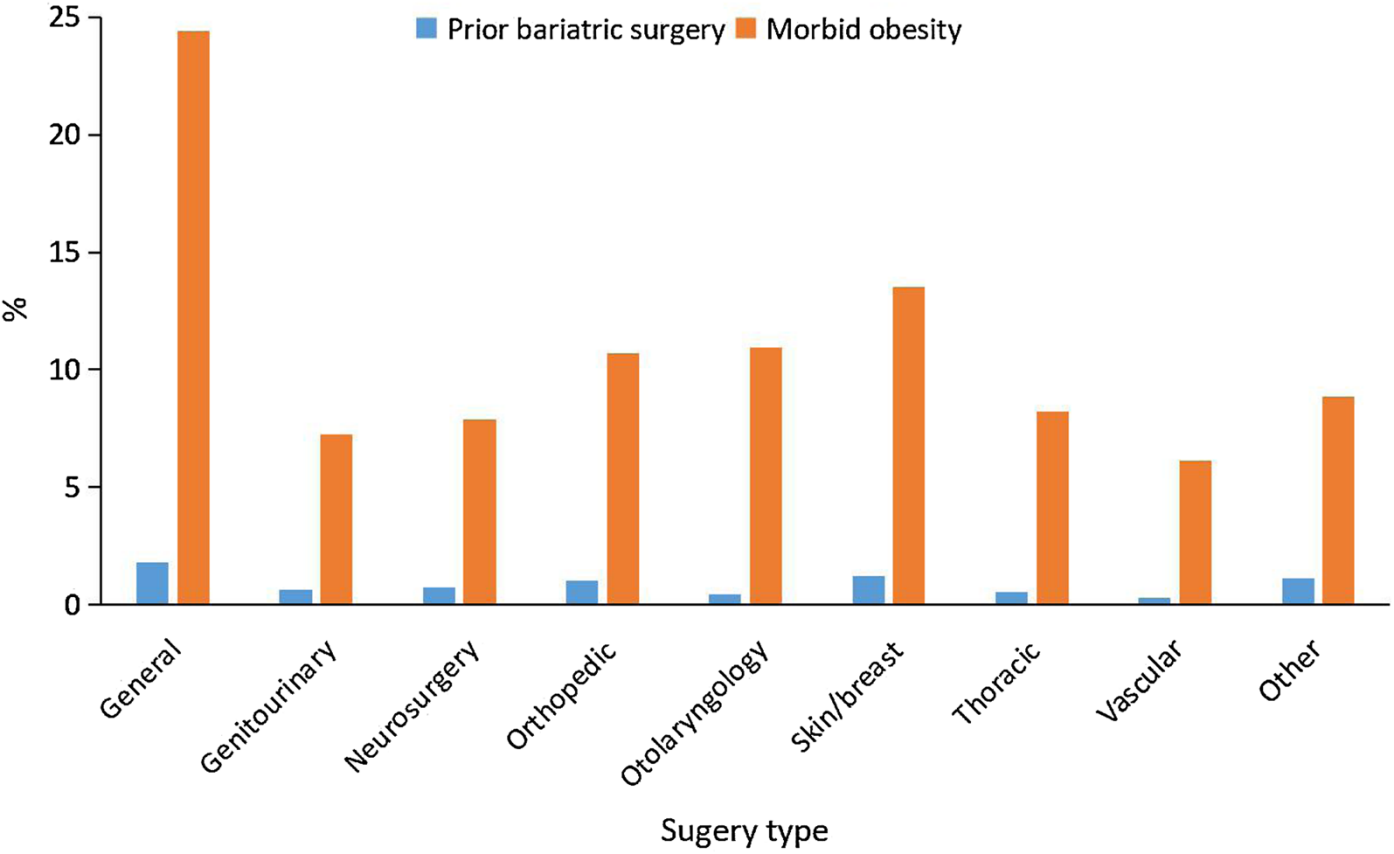
Frequency of bariatric surgery and morbid obesity in patients with type 2 diabetes mellitus by type of noncardiac surgery

From 2006 to 2014, an estimated 1,526,820 adults hospitalized for major noncardiac surgery were identified as T2DM patients, among whom 119,002 (7.79%) were diagnosed with prior-BS, and 1,407,818 (92.21%) were diagnosed with morbid obesity. The proportion of morbid obesity in T2DM patients undergoing noncardiac surgery increased significantly over time (7.42% in 2006 to 17.08% in 2014, P for trend < 0.0001). Additionally, the proportion of patients with prior-BS also increased significantly over time (0.11% in 2006 to 1.55% in 2014, P for trend < 0.0001) (Fig. 1). The BMI changes from 2006 to 2014 in patients with prior-BS were shown in Additional file 1: Table S2, which suggested that the proportions of obesity and morbid obesity in the prior-BS group has significantly increased from 2006 to 2014 (P for trend < 0.0001). Furthermore, rates of prior-BS and morbid obesity were compared among nine categories of surgery. Patients who underwent general surgery had the highest rates for both prior-BS and morbid obesity (1.82% and 24.42%, respectively), followed by skin/breast surgery (1.22% and 13.53%, respectively; Fig. 2).

Patients with prior-BS tended to be younger, female, and white; have lower rates of cardiovascular risk factors (such as dyslipidaemia, hypertension and coronary artery disease); and have better control of diabetes and lower rate of diabetes-related complications (Table 1). However, patients with prior-BS had higher rates of smoking, alcohol abuse and drug abuse. In addition, patients with prior-BS had higher rates of prior cardiovascular surgeries than subjects with morbid obesity. These patients were also more likely to have undergone orthopaedic surgery and less likely to have undergone general surgery (Table 1). Considering BMI categories, all patients with morbid obesity had a BMI of ≥ 40 kg/m^2^ while patients with prior-BS are categorized into three groups: BMI < 30 kg/m^2^, 30 ≤ BMI < 40 kg/m^2^, and BMI ≥ 40 kg/m^2^ and 50.79% of patients with prior-BS had a BMI of < 30 kg/m^2^ (Table 1).

**Table 1:** Comparison of baseline characteristics between prior bariatric surgery and morbid obesity in diabetes mellitus patients undergoing major noncardiac surgery

Variables	Morbid obesity (N = 1,407,818, %)	Prior-BS (N = 119,002, %)	P-value
Mean age (SE)	57.06 (0.08)	56.65 (0.09)	< 0.0001
Female	903,144 (64.15)	85,722 (72.03)	< 0.0001
Race			< 0.0001
White	900,133 (63.94)	82,259 (69.12)	
Black	188,572 (13.39)	12,648 (10.63)	
Hispanic	108,798 (7.73)	7263 (6.10)	
Other	49,465 (3.51)	3353 (2.82)	
Missing	160,850 (11.43)	13,478 (11.33)	
Elective surgery	880,610 (62.55)	70,994 (59.66)	< 0.0001
Smoking	299,815 (21.30)	27,403 (23.03)	< 0.0001
Alcohol abuse	13,173 (0.94)	1358 (1.14)	0.0043
Drug abuse	14,596 (1.04)	1341 (1.13)	0.2402
Dyslipidemia	184,342 (13.09)	11,594 (9.74)	< 0.0001
Hypertension	1,120,839 (79.62)	86,518 (72.70)	< 0.0001
Coronary artery disease	250,654 (17.80)	18,493 (15.54)	< 0.0001
End stage renal disease	211,300 (15.01)	11,105 (9.33)	< 0.0001
Congestive heart failure	156,748 (11.13)	6434 (5.41)	< 0.0001
Chronic lung disease	353,349 (25.10)	24,857 (20.89)	< 0.0001
Chronic liver disease	83,095 (5.90)	4083 (3.43)	< 0.0001
Peripheral vascular disorders	102,994 (7.32)	5490 (4.61)	< 0.0001
Malignancy	28,561 (2.03)	1814 (1.52)	< 0.0001
Anemia	255,997 (18.18)	24,276 (20.40)	< 0.0001
Valvular disease	40,325 (2.86)	3047 (2.56)	0.0068
Prior venous thromboembolism	62,459 (4.44)	7368 (6.19)	< 0.0001
Prior transient ischemic attack/stroke	59,134 (4.20)	5422 (4.56)	0.0171
Prior myocardial infarction	65,683 (4.67)	5638 (4.74)	0.6295
Prior percutaneous coronary intervention	62,215 (4.42)	6625 (5.57)	< 0.0001
Prior coronary artery bypass grafting	53,829 (3.82)	4827 (4.06)	0.1007
Uncontrolled diabetes mellitus	168,409 (11.96)	6070 (5.10)	< 0.0001
Diabetes mellitus with complications	245,276 (17.42)	15,966 (13.42)	< 0.0001
BMI categories			–
BMI < 30 kg/m^2^	–	60,443 (50.79)	
30 ≤ BMI < 40 kg/m^2^	–	19,862 (16.69)	
BMI ≥ 40 kg/m^2^	1,407,818 (100)	38,697 (32.52)	
Surgery type			< 0.0001
General	582,299 (41.36)	44,587 (37.47)	
Genitourinary	45,714 (3.25)	4260 (3.58)	
Neurosurgery	44,539 (3.17)	4419 (3.72)	
Orthopedic	502,649 (35.70)	48,709 (40.93)	
Otolaryngology	8654 (0.61)	360 (0.30)	
Skin/breast	93,878 (6.67)	8655 (7.27)	
Thoracic	16,382 (1.17)	1140 (0.96)	
Vascular	100,429 (7.13)	5104 (4.29)	
Other	13,276 (0.94)	1767 (1.48)	

*SE* standard error, BS bariatric surgery

### Analysis of the prevalence of and trends in MACCEs

**Fig. 3:**
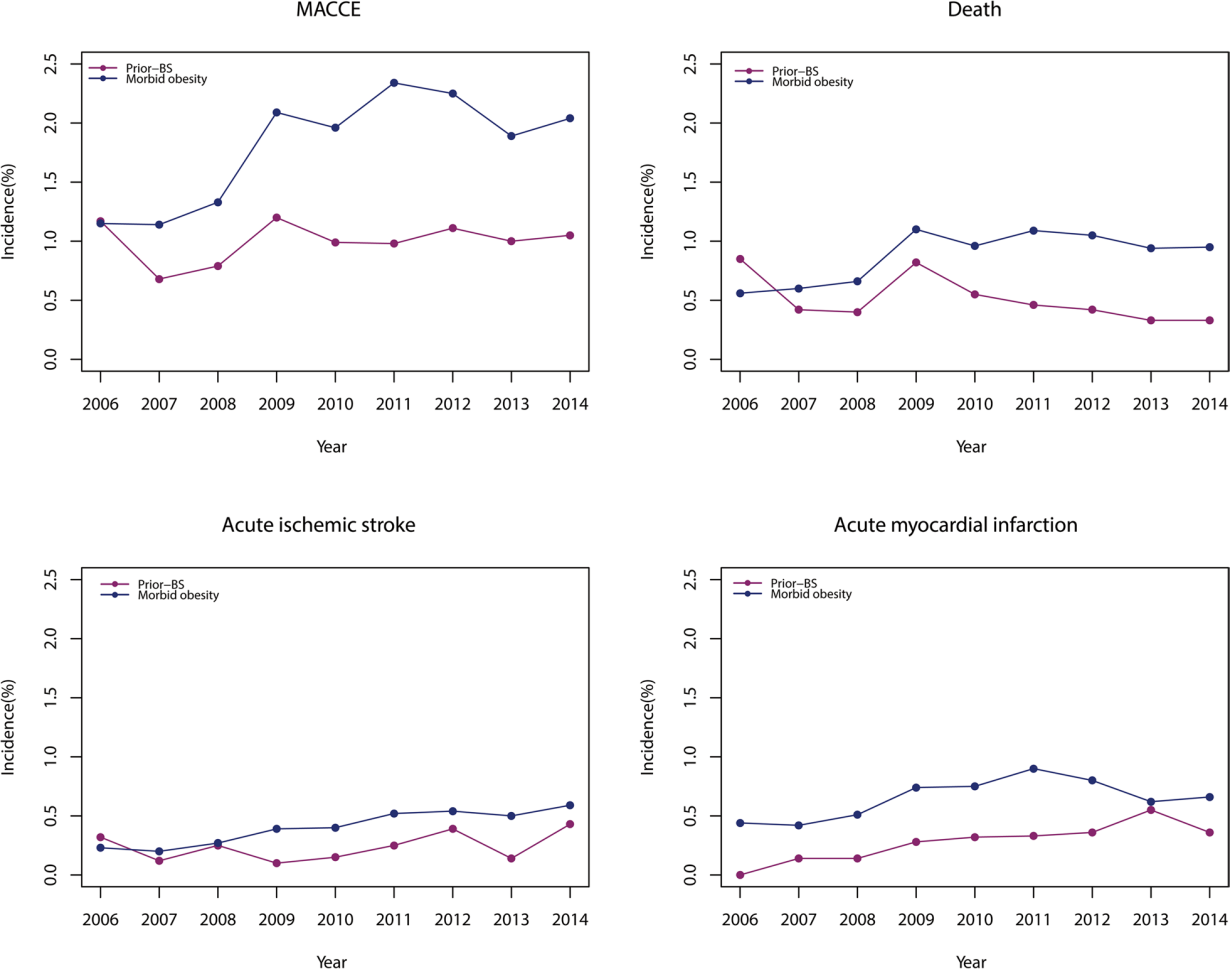
Trends in incidence of perioperative MACCE in type 2 diabetic patients with bariatric surgery and morbid obesity

From 2006 to 2014, the rate of perioperative MACCEs was approximately 1.0% and did not change significantly over time (0.68% to 1.11%, P for trend = 0.0725) among patients with prior-BS, while the rate increased significantly from 1.15 to 2.04% among patients with morbid obesity (P for trend < 0.0001) (Fig. 3). Regarding perioperative death, the rate declined significantly from 0.85% in 2006 to 0.33% in 2014 (P for trend < 0.0001) among patients with prior-BS. In contrast, among patients with morbid obesity, the rate of perioperative death increased significantly over time (0.56% in 2006 to 0.95% in 2014, P for trend < 0.0001) (Fig. 3). The rate of perioperative AIS increased among both patients with prior-BS (0.32% to 0.43%, P for trend < 0.0001) and those with morbid obesity (0.23% to 0.59%, P for trend < 0.0001). Similarly, the rate of perioperative AMI increased among both patients with prior-BS (0.00% to 0.36%, P for trend < 0.0001) and those with morbid obesity (0.44% to 0.66%, P for trend < 0.0001).

### Comparison of perioperative outcomes between patients with prior-BS and morbid obesity

MACCEs occurred in 1210 (1.01%) major noncardiac surgeries among patients with prior BS, compared with 46,225 (3.25%) among patients with morbid obesity (P < 0.0001). Unadjusted analysis suggested that prior-BS was associated with a reduced risk of all perioperative outcomes. After multivariable adjustment, we found that prior-BS was associated with a reduced risk of MACCEs (OR = 0.71; 95% confidence interval [CI] 0.62–0.81), death (OR = 0.64, 95% CI 0.52–0.78), AMI (OR = 0.71, 95% CI 0.57–0.89), acute kidney injury (OR = 0.66, 95% CI 0.62–0.70) and acute respiratory failure (OR: 0.46; 95% CI 0.42–0.50; Table 2). Results from the multivariable analysis did not significantly change after additional adjustment for complications and BMI (Additional file 1: Tables S3, S4). Furthermore, we categorized patients with prior-BS into two groups: BMI < 35 kg/m^2^ and BMI ≥ 35 kg/m^2^ to explore whether the effects were different in prior-BS patients with and without weight loss (BMI < 35 kg/m^2^). The results suggested that the improved outcomes in the prior-BS group were BMI-dependent and more pronounced in patients who had weight loss (Additional file 1: Table S5).

**Table 2:** Comparison of perioperative cardiovascular outcomes between prior bariatric surgery and morbid obesity in diabetes mellitus patients undergoing major noncardiac surgery

Outcomes	Event rates (%)		Logistic regression analysis			
	Prior BS	Morbid obesity	Unadjusted OR (95% CI)	P-value	Adjusted OR (95% CI)^a^	P-value
MACCEs	1.01	3.25	0.52 (0.46, 0.60)	< 0.0001	0.71 (0.62, 0.81)	< 0.0001
Death	0.46	1.52	0.50 (0.41, 0.61)	< 0.0001	0.64 (0.52, 0.78)	< 0.0001
Acute ischemic stroke	0.25	0.90	0.56 (0.43, 0.72)	< 0.0001	0.78 (0.60, 1.01)	0.0559
Acute myocardial infarction	0.33	1.13	0.49 (0.39, 0.61)	< 0.0001	0.71 (0.57, 0.89)	0.0030
Cardiogenic shock	0.04	0.12	0.41 (0.21, 0.79)	0.0077	0.65 (0.33, 1.27)	0.2055
Acute kidney injury	5.83	9.63	0.52 (0.49, 0.55)	< 0.0001	0.66 (0.62, 0.70)	< 0.0001
Acute respiratory failure	2.75	4.69	0.40 (0.37, 0.43)	< 0.0001	0.46 (0.42, 0.50)	< 0.0001

*OR* odds ratio, *CI* confidence interval, *BS* bariatric surgery, *MACCEs* major perioperative adverse cardiovascular and cerebrovascular events

^a^Adjusted for age, sex, race, elective surgery, smoking, alcohol abuse, drug abuse, dyslipidemia, hypertension, coronary artery disease, end stage renal disease, congestive heart failure, chronic lung disease, chronic liver disease, peripheral vascular disorders, malignancy, anemia, valvular disease, prior venous thromboembolism, prior transient ischemic attack/stroke, prior myocardial infarction, prior percutaneous coronary intervention, prior coronary artery bypass grafting, uncontrolled diabetes mellitus and surgery type

Additionally, in order to exclude the influence of bariatric surgery interventions performed during the current hospitalization, we identified bariatric surgery interventions through ICD-9-CM procedural codes and found 4937 (4.15%) and 332,248 (23.60%) patients who underwent bariatric surgery interventions during hospitalization were identified in the prior-BS group and morbid obesity group, respectively (P < 0.0001). We conducted further analysis excluding those who underwent bariatric surgery interventions during hospitalization and the results also suggested lower MACCE and other perioperative complications in the prior-BS group (Additional file 1: Table S6).

### Propensity score and sensitivity analysis

Additional file 1: Table S7 shows the baseline characteristics of patients with prior-BS and morbid obesity before and after 1:1 propensity score matching analysis. All the absolute standardized differences after matching were < 0.1, which indicated acceptable similarity between the two groups. In the matched sample, the incidence of MACCEs was 1.10% and 1.49% in patients with prior-BS and morbid obesity, respectively. Patients in the prior-BS group had lower rates of MACCEs (OR = 0.65, 95% CI 0.53–0.79), death (OR = 0.50, 95% CI 0.37–0.68), AIS (OR = 0.46, 95% CI 0.24–0.90), AMI (OR = 0.63, 95% CI 0.49–0.99), acute kidney injury (OR = 0.62, 95% CI 0.57–0.67) and acute respiratory failure (OR: 0.44; 95% CI 0.40–0.49; Table 3).

**Table 3:** Comparison of perioperative cardiovascular outcomes between prior bariatric surgery and morbid obesity in diabetes mellitus patients undergoing major noncardiac surgery based on propensity score matching analysis

Outcomes	Event rates (%)		Propensity score matching analysis	
	Prior BS	Morbid obesity	Adjusted OR (95% CI)^a^	P-value
MACCEs	1.01	1.49	0.65 (0.53,0.79)	< 0.0001
Death	0.46	0.84	0.50 (0.37, 0.68)	< 0.0001
Acute ischemic stroke	0.25	0.30	0.46 (0.24, 0.90)	0.0222
Acute myocardial infarction	0.33	0.45	0.63 (0.49, 0.99)	0.0431
Cardiogenic shock	0.04	0.05	0.83 (0.45, 1.52)	0.5377
Acute kidney injury	5.83	8.64	0.62 (0.57, 0.67)	< 0.0001
Acute respiratory failure	2.75	5.50	0.44 (0.40, 0.49)	< 0.0001

*OR* odds ratio, *CI* confidence interval, *BS* bariatric surgery, *MACCEs* major perioperative adverse cardiovascular and cerebrovascular events

^a^Adjusted for age, sex, race, elective surgery, smoking, alcohol abuse, drug abuse, dyslipidemia, hypertension, coronary artery disease, end stage renal disease, congestive heart failure, chronic lung disease, chronic liver disease, peripheral vascular disorders, malignancy, anemia, valvular disease, prior venous thromboembolism, prior transient ischemic attack/stroke, prior myocardial infarction, prior percutaneous coronary intervention, prior coronary artery bypass grafting, uncontrolled diabetes mellitus and surgery type

Furthermore, we conducted sensitivity analysis for MACCEs to explore the influence of assumed unmeasured confounders on the robustness of the results. As shown in Additional file 1: Table S8, only if the OR of the hypothetical unmeasured factor reached 2.5 would the result become statistically nonsignificant, which suggested that the lower rate of MACCEs in patients with prior-BS was robust even under strong unmeasured confounders.

## Discussion

In this analysis of 1.5 million major noncardiac surgeries performed on patients with T2DM, MACCEs occurred in 1.01% of patients with prior-BS compared with 3.25% of patients with morbid obesity. The rate of perioperative MACCEs did not change significantly over time (0.68% to 1.11%) among patients with prior-BS due to reductions in perioperative death. In contrast, the rate of MACCEs increased significantly from 1.15 to 2.04% among patients with morbid obesity. Overall, prior-BS was associated with a decreased risk of perioperative MACCEs after multivariable adjustment (OR = 0.71, 95% CI 0.62–0.81). To our knowledge, this is the largest study to report on national trends in cardiovascular outcomes among T2DM patients with morbid obesity undergoing major noncardiac surgery. Our findings suggest a benefit of bariatric surgery in T2DM patients to reduce the risk of perioperative cardiovascular events during noncardiac surgery.

### T2DM, obesity and noncardiac surgery

Patients with obesity and T2DM comprise a large portion of the population undergoing major noncardiac surgery, and the rate is increasing rapidly [5, 17]. The burden of cardiovascular risk factors (including diabetes, obesity, dyslipidaemia and hypertension) and the prevalence of atherosclerotic cardiovascular disease increased over time after analysing 10.5 million patients hospitalized for noncardiac surgery in the US [18]. In the present study, the rate of morbid obesity increased from 7.42% in 2006 to 17.08% in 2014 among T2DM patients undergoing noncardiac surgery, imposing a heavy burden on individuals and health care systems. Surgical patients with T2DM and obesity have more comorbidities than patients without T2DM and obesity. A prospective study included 7565 surgical inpatients and found that diabetes was associated with increased 6-month mortality, major complications and intensive care unit admission [19]. Obesity, accompanied by not only metabolic disorder but also sleep apnoea and hypoventilation syndrome, increased the rate of postoperative respiratory failure, heart failure, prolonged intubation and intensive care unit transfer [20, 21]. A series of observational studies consistently reported that patients with diabetes and obesity have worse postoperative outcomes, including longer operative times and lengths of stay, increased costs and higher rates of infection and readmission [22–25]. The American College of Cardiology/American Heart Association also included diabetes as an important factor in perioperative cardiovascular risk stratification and management for the cardiovascular evaluation and management of patients undergoing noncardiac surgery [26].

### Bariatric surgery and cardiovascular benefits

Bariatric surgery is the most effective method to achieve substantial and durable weight loss in people with obesity. The procedure presents a low risk of complications and morbidity, significantly improving quality of life and overall survival, particularly by reducing death due to cardiovascular disease [27]. Although higher perioperative risk and more adverse events were observed in coronary artery disease (CAD) patients undergoing bariatric surgery than in non-CAD operated patients [28], a recent meta-analysis revealed that bariatric surgery reduced long-term mortality in patients with morbid obesity [29] and reduced morbidity in obese patients above 43 years old [30]. For patients with T2DM, bariatric surgery not only exhibited superiority over medical treatment in achieving improved glycaemic control but also exhibited cardiovascular benefits [10]. Bariatric surgery was reported to reduce hypertension, hyperlipidaemia and cardiovascular risk in T2DM patients [31] and reduce epicardial fat mass and ameliorate atrial fibrillation [32]. The early reduction in circulating follistatin after bariatric surgery predicted the improvement in insulin sensitivity observed later in morbidly obese individuals with and without T2DM [33]. Furthermore, the preventive effect of bariatric surgery on mortality was maintained for up to 23 years, while the effects of bariatric surgery on comorbidities and hospital admissions increased over time [34].

However, the influence of bariatric surgery on cardiovascular events during the perioperative period in patients with T2DM has not been investigated. The present study showed that patients with prior-BS had a significantly lower rate of perioperative MACCEs than those with morbid obesity (1.01% versus 3.25%, P < 0.0001). As previously reported, perioperative MACCEs occurred in 3.0% of the general population [2] and 3.3% of patients with diabetes [11], which was consistent with the results of T2DM patients with morbid obesity in the present analysis. On the other hand, the significantly lower rate of perioperative MACCEs in the prior-BS group indicated a benefit of bariatric surgery on cardiovascular events during the perioperative period, which correlated with better control of diabetes, dyslipidaemia and hypertension, even in the presence of poor control of smoking, alcohol abuse and drug abuse (Table 1). Furthermore, unadjusted analysis suggested that prior-BS was associated with a reduced risk of all perioperative outcomes, but the association with AIS disappeared after multivariable adjustment, suggesting that the effect of bariatric surgery on AIS was dependent on general characteristics, comorbidities, cardiovascular risk factors and events, while bariatric surgery had an independent effect on MACCEs, death and AMI.

### Differences in MACCEs between general and prior-BS diabetic patients

The trends in MACCEs and individual end points among T2DM patients with prior-BS compared with those among T2DM patients with morbid obesity are encouraging. The adjusted percentage change in MACCEs increased by 6% from 2004 to 2013 in diabetic subjects compared with the percent change in those without [11], while the rate of MACCEs doubled from 2006 (1.15%) to 2014 (2.04%) in T2DM patients with morbid obesity. In contrast, the rate of perioperative MACCEs was approximately 1.0% and did not change significantly over time for T2DM patients with prior BS. These data indicated that bariatric surgery may prevent the adverse MACCE effects associated with morbid obesity by resulting in weight loss, reducing cardiovascular risk factors (uncontrolled diabetes, hyperlipemia and hypertension) and facilitating a return to the characteristics associated with general diabetic patients or improving these characteristics even further. Notably, concerning each individual end point, the reduction in perioperative death (0.85% in 2006 to 0.33% in 2014) among patients with prior-BS was consistent with that of general diabetic patients (adjusted percentage decreased by 14%), while the rate increased significantly over time (0.56% in 2006 to 0.95% in 2014) among patients with morbid obesity. A recent retrospective analysis included 431,480 subjects and reported that perioperative glucose predicted 30-day mortality after surgery, which was linear for noncardiac surgery but nonlinear for cardiac procedures [35]. Better glycaemic control, especially during the perioperative period, was a vital factor associated with the perioperative mortality of diabetic subjects. In the present analysis, patients with prior-BS had better diabetes control (94.90% vs. 88.04%) along with a lower rate of dyslipidaemia (9.74% vs. 13.09%) and hypertension (72.70% vs. 79.62%) (Table 1), which explained the reduced perioperative mortality in part.

Owing to improved control of cardiovascular risk factors, the trends in AMI and AIS of US patients with diabetes are improving in nonoperative settings [36], but the trends are inconsistent in operative settings [11]. The unfavourable trends in perioperative cardiovascular outcome may be attributed to platelet activation, catecholamine surges, bleeding and inflammation associated with the surgical procedure [11]. Trends in AMI increased both in the prior-BS and morbid obesity groups of T2DM diabetes in the current study, which contrasted with the adjusted reduction of 7% among general diabetic patients [11]. On the one hand, since there was a lower rate of AMI in the prior-BS group than in the morbid obesity group (0.33% vs. 1.13%), bariatric surgery had a positive influence on perioperative AMI by resulting in weight loss, reducing cardiovascular risk factors, alleviating cardiac workload and altering serum adipokine patterns. On the other hand, the positive influence was still limited, and smoking, alcohol abuse and drug abuse may take part in the increased trend in AMI in T2DM patients with prior-BS (Table 1).

Although the incidence of AIS has declined in the overall US population and among diabetic patients in recent decades [37], the rate still increased in the overall and diabetic populations during the perioperative period [2, 11]. The trend in perioperative AIS among T2DM patients with prior-BS and morbid obesity was consistent with previous observational data, indicating that bariatric surgery was unable to reverse the increasing trend in perioperative AIS in diabetic patients, although it could reduce the perioperative AIS rate compared with that of morbidly obese subjects. The increasing rate of perioperative AIS, both in the overall population and among patients with diabetes and morbid obesity, may be attributable to altered intraoperative haemodynamic management, use of perioperative β blockers and an increasing prevalence of cardiovascular risk factors among surgical patients [37, 38].

There are several limitations in the present study. First, as we used the NIS database, which is based on administrative coding data, reporting bias or coding errors may exist and be ineluctable. Also, since NIS is an inpatient database focusing only on outcomes occurred during hospitalization and information in the NIS database is de-identified for patient privacy, we could not link discharge or long-term outcomes from other sources. Second, the measures of T2DM control and complications are limited, and data on the course of disease, the level of glucose control, C-peptide level, glycosylated haemoglobin, the treatment regimen and insulin use are lacking, which are important predictors of CVD risk for patients with T2DM [11]. In the present study, we adjusted for uncontrolled T2DM and diabetes with complications as a variate in logistic analysis. Third, to avoid mixing T2DM patients with gestational diabetes patients, patients undergoing obstetric and gynaecological surgeries were not included in the present study, which may have excluded some T2DM patients who were not pregnant. Fourth, data on cardiovascular medication, anticoagulants, and perioperative cardiac biomarkers were not available in the NIS database.

## Conclusion

In conclusion, obesity and T2DM were both vital risk factors for CVD and perioperative MACCEs. Prior bariatric surgery in T2DM patients who underwent noncardiac surgery is associated with a lower risk of MACCEs and a lower incidence of postoperative mortality. Bariatric surgery is a promising treatment for obese T2DM patients and not only provides better glycaemic control and sustained weight loss but also reduces the risk of CVD and MACCEs in individuals undergoing noncardiac surgery. Since the current study was observational, prospective studies are needed to verify the benefits of bariatric surgery in noncardiac surgery patients.

## Supplementary information

**Additional file 1: Table S1.** ICD-9-CM codes used in the study. **Table S2.** Changes in BMI categories from 2006 to 2014 in the prior-BS group. **Table S3.** Comparison of outcomes after additionally adjusted for diabetes-related complications. **Table S4.** Comparison of outcomes after adjusted for diabetes-related complications and BMI. **Table S5.** Comparison of outcomes stratified by BMI in the prior-BS group. **Table S6.** Comparison of outcomes between prior bariatric surgery and morbid obesity after excluding patients receiving BS interventions during the current hospitalization. **Table S7.** Baseline characteristics and standardized mean differences between prior bariatric surgery and morbid obesity in diabetes mellitus patients undergoing major noncardiac surgery before and after propensity score matching analysis. **Table S8.** Sensitivity analysis for comparison of MACCEs between prior bariatric surgery and morbid obesity in diabetes mellitus patients undergoing major noncardiac surgery in propensity score matched sample.

## Data Availability

The datasets generated and/or analyzed during the current study are available at https://www.hcup-us.ahrq.gov/databases.jsp.

## Abbreviations

MACCEs: Major adverse cardiovascular and cerebrovascular events
CVD: Cardiovascular diseases
T2DM: Type 2 diabetes mellitus
NIS: National Inpatient Sample
HCUP: Healthcare Cost and Utilization Project
CCS: Clinical Classifications Software
prior-BS: Prior bariatric surgery
AMI: Acute myocardial infarction
AIS: Acute ischemic stroke
ORs: Odds ratios
CI: Confidence interval
