# Supplementary material for: Prior bariatric surgery and perioperative cardiovascular outcomes following noncardiac surgery in patients with type 2 diabetes mellitus: hint from National Inpatient Sample Database
Source: Cardiovasc Diabetol. 2020 Jul 6;19:103. doi: 10.1186/s12933-020-01084-7 (PMC7339406; doi:10.1186/s12933-020-01084-7)
Supplement: Supplementary file 1 — Additional file 1: Table S1. ICD-9-CM codes used in the study. Table S2. Changes in BMI categories from 2006 to 2014 in the prior-BS group. Table S3. Comparison of outcomes after additionally adjusted for diabetes-related complications. Table S4. Comparison of outcomes after adjusted for diabetes-related complications and BMI. Table S5. Comparison of outcomes stratified by BMI in the prior-BS group. Table S6. Comparison of outcomes between prior bariatric surgery and morbid obesity after excluding patients receiving BS interventions during the current hospitalization. Table S7. Baseline characteristics and standardized mean differences between prior bariatric surgery and morbid obesity in diabetes mellitus patients undergoing major noncardiac surgery before and after propensity score matching analysis. Table S8. Sensitivity analysis for comparison of MACCEs between prior bariatric surgery and morbid obesity in diabetes mellitus patients undergoing major noncardiac surgery in propensity score matched sample. [file 12933_2020_1084_MOESM1_ESM.docx]

**Table S1. ICD-9-CM codes used in the study.**

| **Variables** | **Codes** |
| --- | --- |
| **Surgery type** |  |
| General | PRCCS1: 66, 67, 68, 71, 72, 73, 74, 75, 78, 79, 80, 81, 84, 85, 86, 89, 90, 94, 96, 99 |
| Genitourinary | PRCCS1: 101, 103, 104, 106, 109, 112, 113, 114, 115, 118 |
| Neurosurgery | PRCCS1: 1, 2, 3, 5, 6, 9 |
| Orthopedic | PRCCS1: 142, 143, 144, 145, 146, 147, 148, 149, 150, 151, 152, 153, 154, 157, 158, 160, 161, 162, 164 |
| Otolaryngology | PRCCS1: 22, 23, 24, 26, 27, 28, 30, 33, 34 |
| Skin/breast | PRCCS1: 166, 167, 169, 170, 172, 175, 176 |
| Thoracic | PRCCS1: 36, 38, 39, 42 |
| Vascular | PRCCS1: 51, 52, 53, 55, 56, 57, 59, 60, 61 |
| Other | PRCCS1: 10, 12, 105, 231 |
| **Prior bariatric surgery** | V45.86 |
| **Current bariatric surgery** | 43.89, 44.95, 44.93, 44.69, 44.68, 45.91, 44.93, 45.51, 44.39, 44.38, 43.82, 43.7 |
| **Morbid obesity** | V85.4, V85.41, V85.42, V85.43, V85.44, 278.01 |
| **Type 2 diabetes mellitus** | 250.00, 250.02, 250.10, 250.12, 250.20, 250.22, 250.30, 250.32, 250.40, 250.42, 250.50, 250.52, 250.60, 250.62, 250.70, 250.72, 250.80, 250.82, 250.90, 250.92 |
| **Uncontrolled type 2 diabetes mellitus** | 250.02, 250.12, 250.22, 250.32, 250.42, 250.52, 250.62, 250.72, 250.82, 250.92 |
| **Diabetes mellitus with complications** | AHRQ Comorbidities |
| **Comorbidities** |  |
| Smoking | 305.1, V15.82 |
| Dyslipidemia | 272.0, 272.1, 272.2, 272.3, 272.4 |
| Coronary artery disease | 414.0x |
| Prior stroke/transient ischemic attack | V12.54, 438.x |
| Previous percutaneous cororary intervention | V45.82 |
| Previous coronary artery bypass grafting | V45.81 |
| Prior venous thromboembolic event | V12.51, V12.55, 435.5, 435.7 |
| Prior myocardial infarction | 412.x |
| Alcohol abuse | AHRQ Comorbidities |
| Drug abuse | AHRQ Comorbidities |
| Hypertension | AHRQ Comorbidities |
| End stage renal disease | AHRQ Comorbidities |
| Congestive heart failure | AHRQ Comorbidities |
| Chronic lung disease | AHRQ Comorbidities |
| Chronic liver disease | AHRQ Comorbidities |
| Peripheral vascular disorders | AHRQ Comorbidities |
| Malignancy | AHRQ Comorbidities |
| Anemia | AHRQ Comorbidities |
| Valvular disease | AHRQ Comorbidities |
| **Outcomes** |  |
| Death | Indicator “die” in the database. |
| Acute ischemic stroke | 436.x, 437.1, 433.01, 433.11, 433.21, 433.31, 433.81, 433.91, 434.01, 434.11, 434.91 |
| Acute myocardial infarction | 410.01, 410.11, 410.21, 410.31, 410.41, 410.51, 410.61, 410.71, 410.81, 410.91 |
| Cardiogenic shock | 785.51 |
| Acute kindey injury | 584.5, 584.6, 584.7, 584.8, 584.9 |
| Acute respiratory failure | 799.1, 518.4, 518.81, 518.82, 51.884, 786.09, 518.51 |
| MACCEs | Death or acute ischemic stroke or acute myocardial infarction |

Abbreviation: MACCEs, major perioperative adverse cardiovascular and cerebrovascular events.

**Table S2. Changes in BMI categories from 2006 to 2014 in the prior-BS group**

| **BMI categories** | **2006** | **2007** | **2008** | **2009** | **2010** | **2011** | **2012** | **2013** | **2014** | **P for trend** |
| --- | --- | --- | --- | --- | --- | --- | --- | --- | --- | --- |
| BMI<30kg/m^2^ | 68.72 | 60.93 | 59.20 | 53.97 | 51.85 | 49.45 | 48.53 | 47.01 | 45.19 | <0.0001 |
| 30≤BMI<40kg/m^2^ | 12.78 | 15.38 | 13.98 | 14.79 | 16.46 | 16.83 | 17.10 | 18.72 | 17.95 |  |
| BMI≥40kg/m^2^ | 18.50 | 23.69 | 26.82 | 31.24 | 31.69 | 33.72 | 34.37 | 34.27 | 36.85 |  |

**Table S3. Comparison of outcomes after additionally adjusted for diabetes-related complications**

| **Outcomes** | **OR (95%CI)*** | **P-value** |
| --- | --- | --- |
| **MACCEs** | 0.71(0.62,0.81) | <0.0001 |
| **Death** | 0.64(0.52,0.77) | <0.0001 |
| **Acute ischemic stroke** | 0.77(0.60,1.00) | 0.0536 |
| **Acute myocardial infarction** | 0.72(0.57,0.90) | 0.0037 |
| **Cardiogenic shock** | 0.64(0.33,1.25) | 0.1924 |
| **Acute kidney injury** | 0.66(0.62,0.70) | <0.0001 |
| **Acute respiratory failure** | 0.46(0.42,0.50) | <0.0001 |

Abbreviation: OR, odds ratio; CI, confidence interval; BS, bariatric surgery; MACCEs, major perioperative adverse cardiovascular and cerebrovascular events.

***** Adjusted for age, sex, race, elective surgery, smoking, alcohol abuse, drug abuse, dyslipidemia, hypertension, coronary artery disease, end stage renal disease, congestive heart failure, chronic lung disease, chronic liver disease, peripheral vascular disorders, malignancy, anemia, valvular disease, prior venous thromboembolism, prior transient ischemic attack/stroke, prior myocardial infarction, prior percutaneous coronary intervention, prior coronary artery bypass grafting, uncontrolled diabetes mellitus, diabetes-related complications and surgery type.

**Table S4. Comparison of outcomes after adjusted for diabetes-related complications and BMI**

| **Outcomes** | **OR (95%CI)*** | **P-value** |
| --- | --- | --- |
| **MACCEs** | 0.74(0.59,0.93) | 0.0107 |
| **Death** | 0.63(0.45,0.88) | 0.0062 |
| **Acute ischemic stroke** | 0.72(0.44,1.18) | 0.1863 |
| **Acute myocardial infarction** | 0.88(0.61,1.27) | 0.4956 |
| **Cardiogenic shock** | 0.69(0.22,2.16) | 0.5216 |
| **Acute kidney injury** | 0.94(0.86,1.03) | 0.1676 |
| **Acute respiratory failure** | 0.68(0.61,0.76) | <0.0001 |

Abbreviation: OR, odds ratio; CI, confidence interval; BS, bariatric surgery; MACCEs, major perioperative adverse cardiovascular and cerebrovascular events.

***** Adjusted for age, sex, race, elective surgery, smoking, alcohol abuse, drug abuse, dyslipidemia, hypertension, coronary artery disease, end stage renal disease, congestive heart failure, chronic lung disease, chronic liver disease, peripheral vascular disorders, malignancy, anemia, valvular disease, prior venous thromboembolism, prior transient ischemic attack/stroke, prior myocardial infarction, prior percutaneous coronary intervention, prior coronary artery bypass grafting, uncontrolled diabetes mellitus, diabetes-related complications, BMI and surgery type.

**Table S5. Comparison of outcomes stratified by BMI in the prior-BS group**

| **Outcomes** | **Prior BS with BMI<35kg/m^2^**  **(n=75775)** | |  | **Prior BS with BMI≥35kg/m^2^**  **(n=43227)** | |
| --- | --- | --- | --- | --- | --- |
|  | **OR (95%CI)*** | **P-value** |  | **OR (95%CI)*** | **P-value** |
| **MACCEs** | 0.71(0.60,0.83) | <0.0001 |  | 0.71(0.57,0.89) | 0.0027 |
| **Death** | 0.66(0.52,0.83) | 0.0006 |  | 0.59(0.43,0.82) | 0.0018 |
| **Acute ischemic stroke** | 0.76(0.55,1.04) | 0.0903 |  | 0.80(0.51,1.25) | 0.3311 |
| **Acute myocardial infarction** | 0.69(0.52,0.91) | 0.0088 |  | 0.78(0.54,1.13) | 0.1828 |
| **Cardiogenic shock** | 0.65(0.29,1.48) | 0.3061 |  | 0.62(0.20,1.94) | 0.4087 |
| **Acute kidney injury** | 0.52(0.48,0.57) | <0.0001 |  | 0.92(0.84,1.00) | 0.0435 |
| **Acute respiratory failure** | 0.34(0.30,0.38) | <0.0001 |  | 0.66(0.59,0.74) | <0.0001 |

Abbreviation: OR, odds ratio; CI, confidence interval; BS, bariatric surgery; MACCEs, major perioperative adverse cardiovascular and cerebrovascular events.

***** Adjusted for age, sex, race, elective surgery, smoking, alcohol abuse, drug abuse, dyslipidemia, hypertension, coronary artery disease, end stage renal disease, congestive heart failure, chronic lung disease, chronic liver disease, peripheral vascular disorders, malignancy, anemia, valvular disease, prior venous thromboembolism, prior transient ischemic attack/stroke, prior myocardial infarction, prior percutaneous coronary intervention, prior coronary artery bypass grafting, uncontrolled diabetes mellitus, diabetes-related complications and surgery type.

**Table S6. Comparison of outcomes between prior bariatric surgery and morbid obesity after excluding patients receiving BS interventions during the current hospitalization**

| **Outcomes** | **OR (95%CI)*** | **P-value** |
| --- | --- | --- |
| **MACCEs** | 0.65(0.51,0.83) | 0.0004 |
| **Death** | 0.57(0.41,0.80) | 0.0010 |
| **Acute ischemic stroke** | 0.69(0.42,1.14) | 0.1463 |
| **Acute myocardial infarction** | 0.73(0.49,1.10) | 0.1298 |
| **Cardiogenic shock** | 0.66(0.21,2.09) | 0.4847 |
| **Acute kidney injury** | 0.82(0.75,0.90) | <0.0001 |
| **Acute respiratory failure** | 0.65(0.58,0.74) | <0.0001 |

Abbreviation: OR, odds ratio; CI, confidence interval; BS, bariatric surgery; MACCEs, major perioperative adverse cardiovascular and cerebrovascular events.

***** Adjusted for age, sex, race, elective surgery, smoking, alcohol abuse, drug abuse, dyslipidemia, hypertension, coronary artery disease, end stage renal disease, congestive heart failure, chronic lung disease, chronic liver disease, peripheral vascular disorders, malignancy, anemia, valvular disease, prior venous thromboembolism, prior transient ischemic attack/stroke, prior myocardial infarction, prior percutaneous coronary intervention, prior coronary artery bypass grafting, uncontrolled diabetes mellitus, diabetes-related complications, BMI and surgery type.

**Table S7. Baseline characteristics and standardized mean differences between prior** **bariatric surgery and morbid obesity in diabetes mellitus patients undergoing major noncardiac surgery before and after propensity score matching analysis**

| **Variables** | **Before propensity score matching** | | |  | **After propensity score matching** | | |
| --- | --- | --- | --- | --- | --- | --- | --- |
|  | **Prior-BS**  **(%)** | **Morbid obesity (%)** | **Standardized difference** |  | **Prior-BS**  **(%)** | **Morbid obesity (%)** | **Standardized difference** |
| Mean age, year | 57.06 | 56.65 | -0.0164 |  |  |  | -0.0055 |
| Female | 64.15 | 72.03 | 0.1697 |  | 71.98 | 72.03 | 0.0011 |
| Race |  |  | 0.1258 |  |  |  | 0.0183 |
| White | 63.94 | 69.12 |  |  | 869.24 | 69.12 |  |
| Black | 13.39 | 10.63 |  |  | 11.01 | 10.63 |  |
| Hispanic | 7.73 | 6.10 |  |  | 65.83 | 6.10 |  |
| Other | 3.51 | 2.82 |  |  | 2.69 | 2.82 |  |
| Missing | 11.43 | 11.33 |  |  | 11.24 | 11.33 |  |
| Elective | 62.55 | 59.66 | -0.0594 |  | 58.22 | 59.66 | 0.0292 |
| Smoking | 21.30 | 23.03 | 0.0417 |  | 21.76 | 23.03 | 0.0304 |
| Alcohol abuse | 0.94 | 1.14 | 0.0203 |  | 0.91 | 1.14 | 0.0229 |
| Drug abuse | 1.04 | 1.13 | 0.0087 |  | 0.94 | 1.13 | 0.0183 |
| Dyslipidemia | 13.09 | 9.74 | -0.1055 |  | 9.36 | 9.74 | 0.0131 |
| Hypertension | 79.62 | 72.70 | -0.1627 |  | 73.22 | 72.70 | -0.0117 |
| Coronary artery disease | 17.80 | 15.54 | -0.0608 |  | 13.97 | 15.54 | 0.0441 |
| End stage renal disease | 15.01 | 9.33 | -0.1743 |  | 9.20 | 9.33 | 0.0045 |
| Congestive heart failure | 11.13 | 5.41 | -0.2091 |  | 5.40 | 5.41 | 0.0001 |
| Chronic lung disease | 25.10 | 20.89 | -0.1002 |  | 20.41 | 20.89 | 0.0118 |
| Chronic liver disease | 5.90 | 3.43 | -0.1174 |  | 2.83 | 3.43 | 0.0342 |
| Peripheral vascular disorders | 7.32 | 4.61 | -0.1143 |  | 4.16 | 4.61 | 0.0220 |
| Malignancy | 2.03 | 1.52 | -0.0382 |  | 1.35 | 1.52 | 0.0149 |
| Anemia | 18.18 | 20.40 | 0.0562 |  | 19.68 | 20.40 | 0.0180 |
| Valvular disease | 2.86) | 2.56 | -0.0187 |  | 2.30 | 2.56 | 0.0167 |
| Prior venous thromboembolism | 4.44 | 6.19 | 0.0783 |  | 5.62 | 6.19 | 0.0243 |
| Prior transient ischemic attack/stroke | 4.20 | 4.56 | 0.0174 |  | 4.03 | 4.56 | 0.0258 |
| Prior myocardial infarction | 4.67 | 4.74 | 0.0034 |  | 3.88 | 4.74 | 0.0423 |
| Prior percutaneous cororary intervention | 4.42 | 5.57 | 0.0527 |  | 4.88 | 5.57 | 0.0308 |
| Prior coronary artery bypass grafting | 3.82 | 4.06 | 0.0119 |  | 3.41 | 4.06 | 0.0340 |
| Uncontrolled diabetes mellitus | 11.96 | 5.10 | -0.2475 |  | 5.25 | 5.10 | -0.0067 |
| Surgery type |  |  | 0.1791 |  |  |  | 0.0372 |
| General | 41.36 | 37.47 |  |  | 36.37 | 37.47 |  |
| Genitourinary | 3.25 | 3.58 |  |  | 3.59 | 3.58 |  |
| Neurosurgery | 3.17 | 3.72 |  |  | 3.50 | 3.72 |  |
| Orthopedic | 35.70 | 40.93 |  |  | 42.29 | 40.93 |  |
| Otolaryngology | 0.61 | 0.30 |  |  | 0.32 | 0.30 |  |
| Skin/breast | 6.67 | 7.27 |  |  | 7.39 | 7.27 |  |
| Thoracic | 1.17 | 0.96 |  |  | 0.85 | 0.96 |  |
| Vascular | 7.13 | 4.29 |  |  | 4.41 | 4.29 |  |
| Other | 0.94 | 1.48 |  |  | 1.28 | 1.48 |  |

Abbreviation: BS, bariatric surgery.

**Table S8. Sensitivity analysis for comparison of MACCEs between prior** **bariatric surgery and morbid obesity in diabetes mellitus patients undergoing major noncardiac surgery in propensity score matched sample**

|  | **Odds ratio for association of a hypothetical unmeasured factor with prior-BS** | | | | | | | | |
| --- | --- | --- | --- | --- | --- | --- | --- | --- | --- |
| **Odds ratio for association of a hypothetical unmeasured factor with morbid obesity** |  | 1.00 | 1.25 | 1.50 | 1.75 | 2.00 | 2.25 | 2.50 | 2.75 |
|  | 1.00 | 2.2e-16 | 2.2e-16 | 2.2e-16 | 2.2e-16 | 2.2e-16 | 2.2e-16 | 2.2e-16 | 2.2e-16 |
|  | 1.25 | 2.2e-16 | 2.2e-16 | 2.2e-16 | 2.2e-16 | 2.2e-16 | 2.2e-16 | 1.5e-15 | 9.1e-15 |
|  | 1.50 | 2.2e-16 | 2.2e-16 | 2.2e-16 | 2.7e-14 | 3.1e-12 | 1.2e-10 | 2.3e-09 | 2.5e-08 |
|  | 1.75 | 2.2e-16 | 2.2e-16 | 2.7e-14 | 5.1e-11 | 1.2e-08 | 6.6e-07 | 1.4e-05 | 0.0001 |
|  | 2.00 | 2.2e-16 | 2.2e-16 | 3.1e-12 | 1.2e-08 | 3.3e-06 | 0.0002 | 0.0025 | 0.0171 |
|  | 2.25 | 2.2e-16 | 2.2e-16 | 1.2e-10 | 6.6e-07 | 0.0002 | 0.0057 | 0.0528 | 0.2044 |
|  | 2.50 | 2.2e-16 | 1.5e-15 | 2.3e-09 | 1.4e-05 | 0.0025 | 0.0528 | 0.2746 | 0.6134 |
|  | 2.75 | 2.2e-16 | 9.1e-15 | 2.5e-08 | 0.0001 | 0.0171 | 0.2044 | 0.6134 | 0.8960 |

Abbreviation: MACCEs, major perioperative adverse cardiovascular and cerebrovascular events.
